# Summer Medical Instruction

**Published:** 1836

**Authors:** 


### VI.—Summer Medical Instruction.

Since the establishment of a hospital, which constantly contains a variety of patients, and is designed as a clinical school for the pupils of the Cincinnati Collоge, the professors of that institution have resolved not to deliver courses of summer lectures. From the first of March to the first of November, every day except Sundays, will be a prescribing day, when the medical and surgical attendants will deliver, in the hospital, such clinical remarks as the different cases may prompt; and twice a week each of them will deliver a lecture, in the medical library room of the College, on the prominent maladies that are under treatment at the time, or have lately terminated. Thus the student who attends regularly, will be present through 200 prescribing hours, and at about 130 Clinical Lectures. In connexion with this, he can have the use of more than a thousand volumes of choice medical books. The expense of these advantages, to him who may matriculate as a pupil of the Cincinnati College, will be as follows: matriculation (not to be repeated in the fall when the lectures commence) two dollars—hospital ticket five dollars—library ticket five dollars—equal to twelve. Should he not matriculate the hospital ticket will be ten, the library five, equal to fifteen. As boarding and lodging can be obtained throughout the vacation for two dollars and fifty cents, or even two dollars and twenty-five cents a week, the aggregate cost of all the advantages here enumerated, need not exceed one hundred dollars for the eight months. We presume that equal opporunities for so low a sum, have not often been presented to students of medicine.

